# Microstructural and Impact Resistance Optimization of Concrete Composites with Waste-Based Aggregate Substitutions

**DOI:** 10.3390/polym17192574

**Published:** 2025-09-23

**Authors:** Maksymilian Stępczak, Mikołaj Kazimierczak, Maciej Roszak, Adam Kurzawa, Krzysztof Jamroziak

**Affiliations:** 1 Department of Mechanics, Materials and Biomedical Engineering, Faculty of Mechanical Engineering, Wroclaw University of Science and Technology, Smoluchowskiego 25 Str., 50-370 Wroclaw, Poland; mikolaj.kazimierczak@pwr.edu.pl (M.K.); maciej.roszak@pwr.edu.pl (M.R.); krzysztof.jamroziak@pwr.edu.pl (K.J.); 2 Department of Lightweight Elements Engineering, Foundry and Automation, Faculty of Mechanical Engineering, Wroclaw University of Science and Technology, Smoluchowskiego 25 Str., 50-370 Wroclaw, Poland; adam.kurzawa@pwr.edu.pl

**Keywords:** low-carbon concrete, dynamic testing, compressive strength, rubber granulate, copper slag, fine glass, polypropylene, SEM analysis, CO_2_ emissions

## Abstract

In the context of growing challenges related to the safety and durability of civil infrastructure, the demand for concrete composites capable of withstanding dynamic and impact loading is steadily increasing. Conventional concrete, owing to its brittle nature and limited energy absorption capacity, does not always meet the performance requirements imposed on protective structures. The construction sector’s substantial contribution to CO_2_ emissions further underscores the need for environmentally responsible solutions. This study therefore explores the effects of partially replacing natural aggregate with waste-derived constituents such as SBR rubber granulate, copper slag, polypropylene and glass granulate on the mechanical properties and impact resistance of concrete. Scanning electron microscopy (SEM) and stereoscopic microscopy were used to characterize the additives’ geometry and interfacial bond quality, providing deeper insight into cement paste–aggregate interactions. Compressive testing confirmed that introducing the recycled components does not preclude meeting essential strength criteria, whereas impact experiments revealed pronounced differences in failure mode, crack propagation, and the specimen’s ability to dissipate kinetic energy. The experimental program was complemented by a life cycle assessment (LCA) that quantitatively estimated the CO_2_ emissions associated with producing each mixture. The findings demonstrate that judiciously selected waste materials can reduce the consumption of virgin resources, enhance concrete functionality, and improve their protective performance, thereby advancing the principles of a circular economy.

## 1. Introduction

Contemporary built environments—including buildings and their attendant technical infrastructure—demand substantial material and energy flows for both construction and operation [1,2]. These activities are far from environmentally neutral; they contribute to global ecological challenges such as climate change. A principal driver of this impact is carbon dioxide (CO_2_) emission, whose major sources in the construction sector are cement manufacture and the extraction and processing of aggregates [3,4,5]. Numerous studies report that waste streams such as end-of-life tires, glass, and metallurgical slags require complex and costly recycling routes [6,7,8]. Consequently, applications that enable their large-scale secondary use—foremost among them construction—are of particular relevance. Consuming billions of tons of mineral resources annually, the sector offers an unparalleled opportunity to implement circular-economy principles.

The construction industry accounts for more than 23% of global CO_2_ emissions, a significant fraction of which stems directly from building materials such as concrete [9]. This situation makes the search for low-emission material alternatives a priority in current engineering and environmental research. Accordingly, the concepts of the circular economy and sustainable construction are gaining strategic importance [10,11,12,13]. European Union waste-management directives explicitly mandate waste prevention, reuse, recovery, and safe disposal [14]. Especially critical is the reuse of secondary raw materials capable of substituting for conventional counterparts, thereby curbing both natural resource depletion and greenhouse gas emissions. One promising pathway involves employing industrial solid wastes as partial replacements for natural aggregate in concrete mixes [15,16,17,18,19,20]. Owing to its heterogeneous structure and compositional flexibility, concrete accommodates such substitutions without altering production technology, enabling the development of “low-carbon concretes” [21,22,23] that retain the requisite mechanical performance while lowering the production-stage carbon footprint.

The demand for materials with extended functionality now goes beyond environmental considerations alone. Escalating armed conflicts have created an urgent need for solutions that enhance the safety of civilian populations and critical infrastructure. Present approaches include additional reinforcement using steel bars, high-strength fiber layers, and various particulate fillers [24,25,26,27]. In the face of threats posed by munitions, artillery, and modern combat drones, it has become imperative to engineer construction materials with improved resistance to dynamic actions, including high speed impacts [28,29,30,31]. Research on concrete in which conventional aggregate is partially replaced by recovered materials thus acquires new strategic significance. After appropriate processing and cleaning, industrial wastes can not only reduce CO_2_ emissions and raw material consumption but also potentially enhance concrete resistance to impact loads owing to the favorable mechanical properties of certain admixtures. For example, synthetic rubbers can improve the material’s energy absorption capacity [32,33,34], whereas glass and metallurgical granulates can refine the composite microstructure, thereby strengthening its integrity [35,36,37,38].

This study presents an experimental and environmental evaluation of concretes in which part of the natural aggregate is replaced with industrial waste (SBR rubber, glass, slag and polypropylene). The investigation covers both the mixtures’ resistance to dynamic loading and their CO_2_ emissions. The experimental program comprised compressive strength tests, microstructural assessment using stereoscopic and scanning electron microscopy (SEM), and impact resistance tests in accordance with STANAG 2280. A complementary life cycle assessment (LCA) quantified the influence of the selected additives on the carbon footprint of the resulting concrete composites.

## 2. Materials and Methods

### 2.1. Materials Used in Concrete Mixes

#### 2.1.1. Cement

Portland cement of strength class CEM I 42.5 R supplied by Odra Opole (Opole, Poland) was used to cast the specimens.

#### 2.1.2. Fine Aggregate

The fine aggregate consisted primarily of locally sourced sand with a particle size of 0–2 mm.

#### 2.1.3. Superplasticizer

Pantahrit RC670 was added as a superplasticizer to improve mix workability and to enhance the cohesion of the concrete matrix.

#### 2.1.4. Copper Slag

Copper slag (Manufacturer: Stanisław Kos, Przedsiębiorstwo Produkcyjno-Usługowo-Handlowe “KOS”, Koło, Poland) with a grain size of 0.4 to 1.4 mm, obtained as a by-product of pyrometallurgical copper smelting, was employed as a fine aggregate. The slag exhibited irregular, sharp-edged particles of high hardness, increasing the contact surface with the cement paste and potentially improving adhesion within the interfacial transition zone. Owing to its natural composition, dominated by silicon and iron oxides, the material showed properties comparable to conventional natural aggregate while offering the environmental advantage of repurposing an industrial waste. Before use, the slag was dried and sieved to ensure granulometric uniformity.

#### 2.1.5. Glass Granulate

Glass granulate (Manufacturer: Stanisław Kos, Przedsiębiorstwo Produkcyjno-Usługowo-Handlowe “KOS”, Koło, Poland) with a particle size of 0.9 to 1.5 mm, produced by mechanical crushing of waste container glass, was used as an inert fine aggregate with potential pozzolanic activity due to its high amorphous silica content and chemical stability. The semi-transparent grains possessed relatively smooth surfaces, which could reduce adhesion to the cementitious matrix; however, this drawback was offset by their geometric uniformity and favorable bulk density characteristics.

#### 2.1.6. SBR Rubber

Styrene-butadiene rubber (SBR) (Unirubber, Węgliniec, Poland) aggregate of 2 to 3 mm, obtained by mechanical recycling of end-of-life car tires, served as an elastic polymer aggregate. Its principal component is SBR, a copolymer of styrene and butadiene. The granulate features low density, high abrasion resistance, and durability under atmospheric ageing. The irregularly shaped grains exhibit resilient structures and porous surfaces that distinguish them from conventional mineral aggregates. In addition to synthetic rubber, the material contains residual additives such as carbon black, plasticizers, and thermal stabilizers retained from the shredding process. This secondary raw material possesses stabilized physicochemical properties and is suitable for engineering applications as a non-absorptive or elastic component in cement-based composites.

#### 2.1.7. Polypropylene

Polypropylene (PP) aggregate of 2 to 3 mm, obtained by mechanical recycling of post-consumer plastic products, served as a lightweight polymer aggregate. It is a thermoplastic polymer formed by the polymerization of propylene. The aggregate features low density, high chemical resistance, and durability under environmental exposure. The irregularly shaped flakes exhibit smooth yet rigid structures and non-porous surfaces that distinguish them from conventional mineral aggregates. In addition to the polymer matrix, the material may contain residual additives such as stabilizers, pigments, and processing aids retained from the manufacturing or recycling process.

#### 2.1.8. Concrete Mixtures

Five concrete mixtures were produced for the low-carbon concrete study: a control mix, FGC (Fine Glass Concrete), CSC (Copper Slag Concrete), RC (Rubber Concrete) and PC (Polypropylene Concrete) (Table 1). The substitution of sand ratio of 10% was selected based on previous studies on rubberized and waste-aggregate concrete, which showed that replacement levels below 5% do not produce measurable changes in the microstructure or impact resistance, whereas levels above 15% lead to a decrease in compressive strength, elastic modulus, and overall durability. The intermediate range 5–15% is commonly identified as a compromise between strength retention and energy absorption capacity. Therefore, a substitution level of 10% was deliberately adopted in this study as a representative value that allows observable effects while maintaining an acceptable residual load-bearing capacity. All specimens were cured in a controlled laboratory environment at 20 ± 2 °C, relative humidity ≥ 95%, for 28 days before testing.

The mixes differed only in the type of fine-aggregate substitute employed, while the proportions of the remaining constituents were held constant. This approach enabled a direct comparison of how each replacement affected the material’s mechanical properties and high impact resistance (Table 2).

### 2.2. Methodology of Investigation

#### 2.2.1. Scanning Electron Microscopy of Aggregates

The aggregate surfaces and elemental compositions were examined with a HITACHI TM-3000 scanning electron microscope (Tokyo, Japan) equipped with an EDS⁄EDX detector. The principal objective was to characterize particle geometry. Images were acquired in secondary-electron mode at an accelerating voltage of 15 kV.

#### 2.2.2. Stereoscopic Microscopy of the Concrete Structure

The manufactured specimens were inspected with an OptaTech SK stereoscopic microscope (OptaTech, Warsaw, Poland) to evaluate sample quality, verify homogeneity and observe the bonding between the aggregate and the cement paste.

#### 2.2.3. Static Compression Test

**Figure 1:**
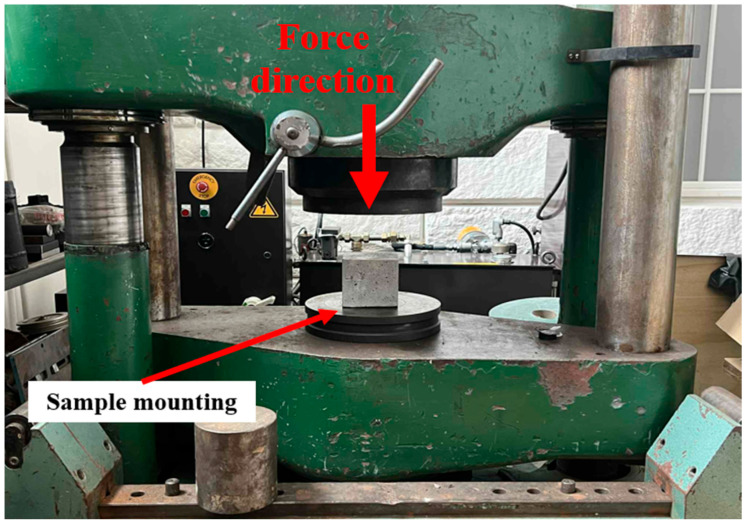
Control specimen subjected to the static compression test.

**Figure 2:**
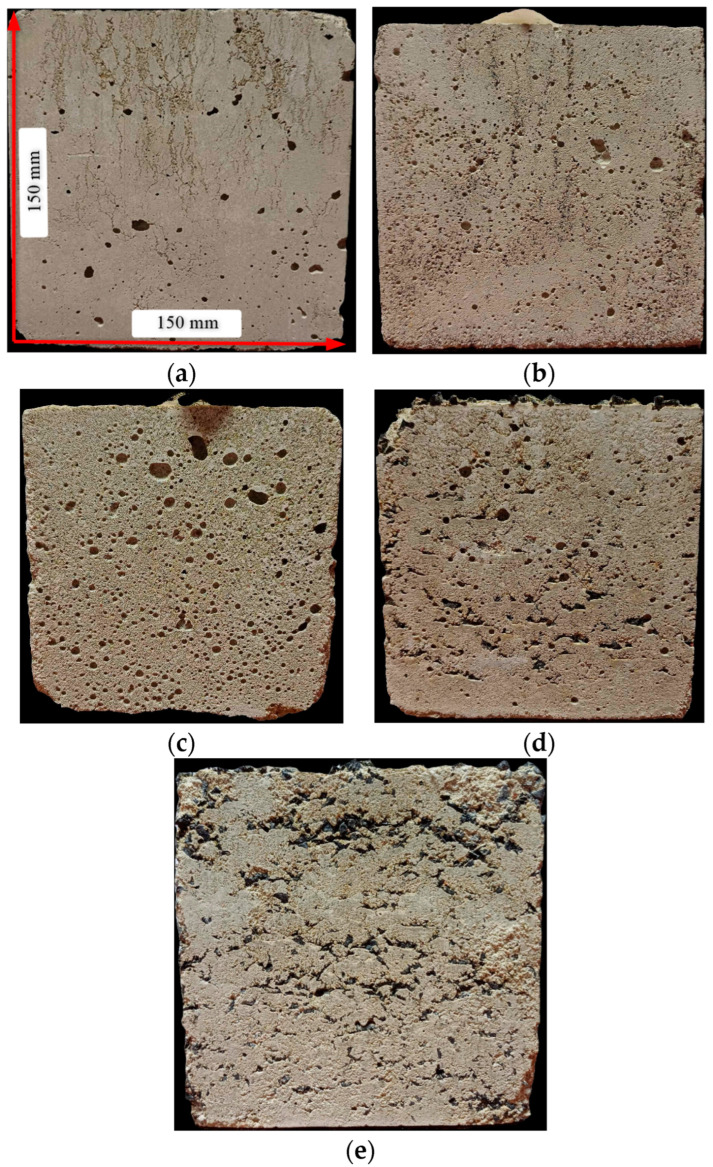
Fabricated specimens: (**a**) control mix, (**b**) CSC, (**c**) FGC, (**d**) RC, (**e**) PC.

The mixtures were evaluated for compressive strength, considered the primary parameter characterizing the composite. Testing followed PN-EN 12390-3:2011/AC:2012, “Testing hardened concrete, Part 3: Compressive strength of test specimens” [39]. The specimens subjected to static compression were cubes measuring 10 × 10 × 10 cm (Figure 1 and Figure 2).

Changes in specimen morphology were observed as the sand replacement aggregate was varied. Specimens containing copper slag exhibited the closest resemblance to the control cubes, whereas the most pronounced differences occurred in mixtures incorporating glass granulate, polypropylene and SBR rubber. Cubes with glass granulate were markedly more porous, indicating water interaction behavior that differs substantially from that of conventional sand.

The density of SBR rubber, in turn, promotes its accumulation in distinct layers. By maintaining an appropriate mixture density, sedimentation can be mitigated and the rubber granulate can be distributed evenly throughout the specimen volume.

Samples containing polypropylene exhibited unstable geometry due to the formation of visible layers of polypropylene flakes throughout the sample volume. This led to insufficient cohesion, pronounced interfacial instability between the constituent materials, and significant inhomogeneity within the structure. As a result, the polypropylene samples showed a high degree of variability and were excluded from impact testing.

**Figure 3:**
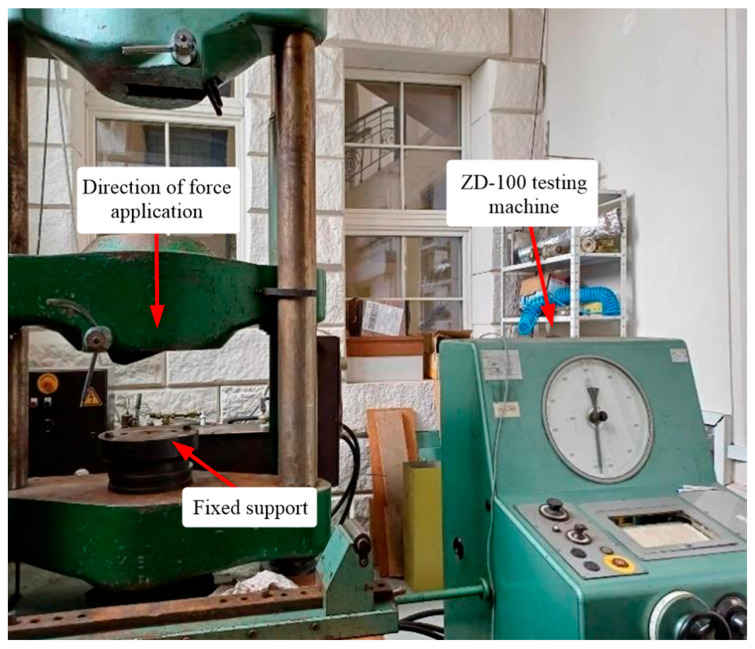
ZD-100 universal testing machine.

The static compression test was carried out on a ZD-100 universal testing machine (VEB Werkzeugmaschinenkombinat “Fritz Heckert”, Betrieb Rauenstein, Leipzig, Germany) (Figure 3) at a force increment rate of 0.6 MPa s^−1^. Specimens were loaded until failure or until the increase in load visibly ceased.

#### 2.2.4. Impact Testing

High impact resistance was evaluated according to STANAG 2280 threat level A, which defines the optimum concrete thickness for small arms ammunition [31]. The impactor types employed are listed in Table 3.

**Figure 4:**
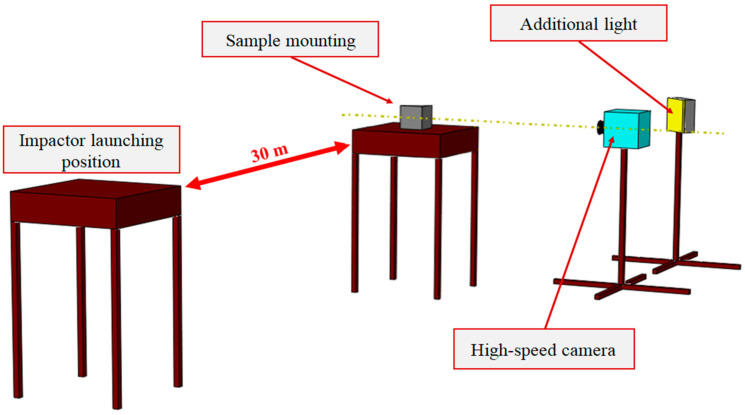
Schematic of the firing-range set-up.

Shooting was performed from a distance of 30 m in compliance with STANAG 2280 (for 9 mm impactor size it was 5 m). The test set-up included a Phantom VEO 710L high-speed camera (Vision Research, Wayne, NJ, USA) that captured the bullet–target interaction and allowed determination of residual bullet velocities after perforation (Figure 4). The principal assessment criterion was whether the impactor penetrated the specimen. Weather conditions during testing were clear skies, no wind and an ambient temperature of 30 °C.

## 3. Results

### 3.1. SEM Analysis Results

**Figure 5:**
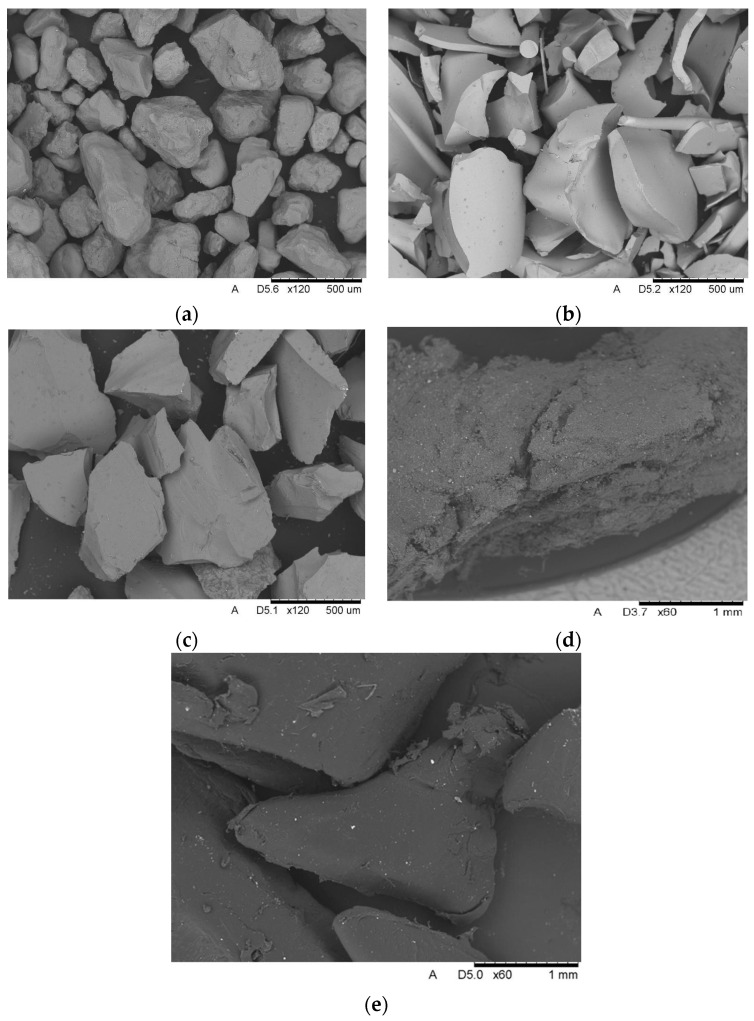
Microscopic images: (**a**) sand, ×120; (**b**) copper slag, ×120; (**c**) glass granulate, ×120; (**d**) SBR rubber, ×60, (**e**) polypropylene petals, ×60.

Scanning electron microscopy was employed to characterize the geometry of the aggregates used in the study. Representative micrographs for each aggregate type are presented in Figure 5. The reference sand exhibits a homogeneous, rounded morphology with particle sizes ranging from 0.2 to 2 mm, consistent with the supplier’s specification. Copper slag shows a broad distribution in both shape and size; no single geometry prevails, as particles with sharp edges coexist with grains displaying more rounded contours. The manufacturer reports a particle size of 0.2 to 0.8 mm, which matches the observed micrographs. Although the morphology of glass granulate resembles that of sand more closely than that of copper slag, its overall particle size is larger, varying from 0.3 to 0.6 mm. The glass particles also possess relatively sharper edges. SBR rubber granulate is the course of the aggregates examined, with particles measuring approximately 2 to 3 mm. Its geometry, as well as that of the polypropylenes, is irregular and frayed, yet it displays the most rounded contours among the aggregates investigated.

Angular aggregate particles enhance concrete strength by promoting more effective interlocking within the binder. This mechanical interlock increases the material’s resistance to micro-crack propagation and improves the composite’s structural integrity. Accordingly, the more “aggressive” the particle geometry, the better the aggregate can bond with the cementitious matrix.

### 3.2. Results of Stereoscopic Microscopy

**Figure 6:**
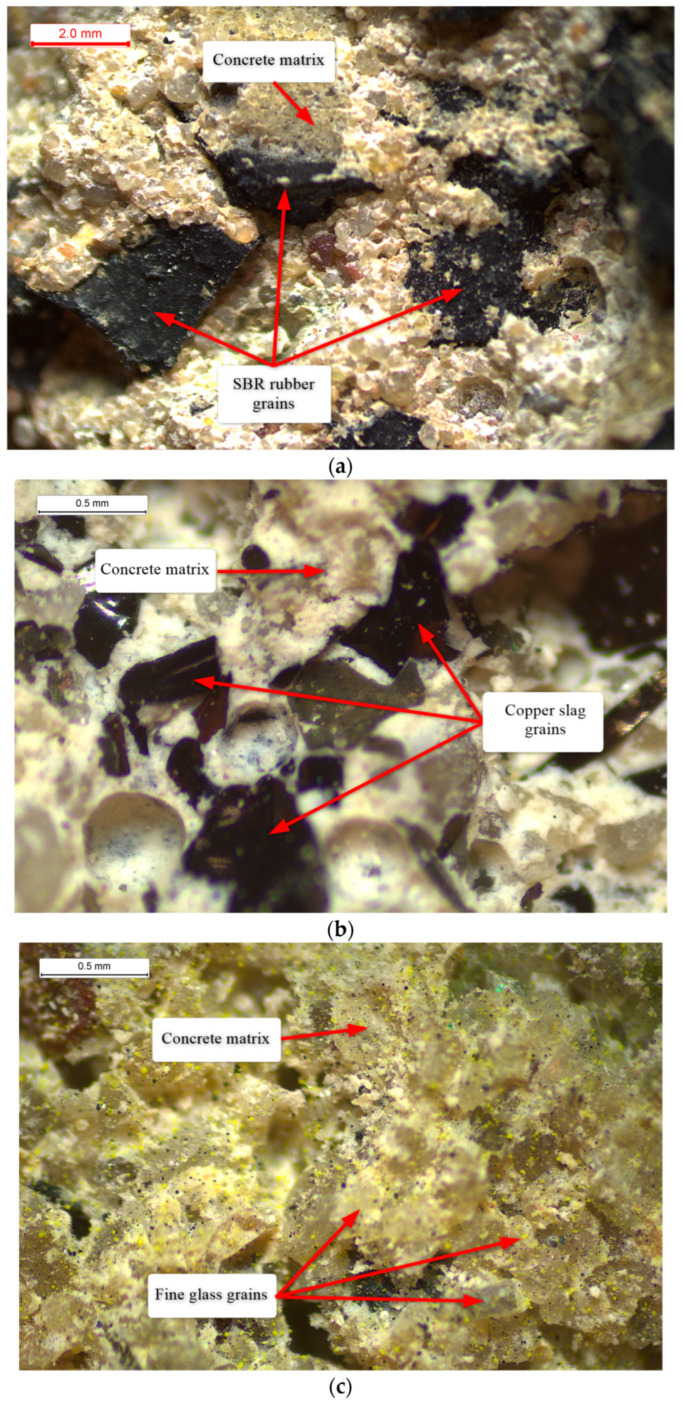
Microscopic images: (**a**) RC specimen, ×20; (**b**) CSC specimen, ×45; (**c**) FGC specimen, ×4.

To clarify how the modified aggregates bond to the cementitious matrix, stereoscopic microscopy was performed on the RC, CSC and FGC series. Images acquired at several magnifications enabled a detailed assessment of the interfacial morphology (Figure 6).

In the RC mixture, SBR rubber particles were significantly larger than the conventional fine aggregate present in the paste. The grains showed rounded, resilient shapes and smooth, slightly porous surfaces. Although the cement paste enveloped the rubber uniformly, mechanical interlocking was absent, which limited adhesion and weakened the interfacial transition zone. This configuration promoted micro-void formation and lowered the composite load-bearing capacity at the macroscale.

The CSC specimen containing copper slag displayed clear interlocking between the irregular, sharp-edged particles and the surrounding cement paste. The hardness and geometry of the slag favored mechanically stable connections, resulting in good structural cohesion. Because the slag particles are similar in size to standard sand, their incorporation did not disturb the composite microstructure.

FGC samples exhibited comparably favorable integration with the cement paste. Glass grains, though somewhat smoother, possessed sharp edges and varied geometries that promoted mechanical anchorage within the matrix. Microscopy revealed effective phase consolidation, despite local areas of reduced wettability that may indicate isolated zones of weaker adhesion. Overall, the interface demonstrated efficient bonding between glass and cement.

Enhancing the bond between rubber and cement paste appears critical. Using a finer rubber fraction and applying a surface treatment, for example, a chemical bath or mechanical roughening, should increase surface roughness and wettability of the rubber particles. These measures are expected to improve adhesion and cohesion across the interfacial transition zone.

### 3.3. Compressive-Strength Results

The results clearly demonstrate that both the reference concrete and the mix containing 30% copper slag (CSC) achieved comparable compressive strengths of approximately 18 MPa, confirming that the hard, angular slag particles do not weaken the material structure. Replacing part of the sand with glass granulate (FGC) reduced strength moderately to about 11 MPa; this decline is linked to the smooth, non-porous glass surface, which limits adhesion and promotes debonding within the interfacial transition zone. The lowest strength values, only 5–6 MPa, were recorded for the SBR rubber concrete (RC) and polypropylene concrete (PC) ex aequo. This marked decrease arises from the rubber’s low elastic modulus and poor particle adhesion: the mechanically inferior phase reduces the effective load-bearing cross-section, leading to local stress concentrations and premature crack initiation. Photographic evidence supports these conclusions. The reference and CSC specimens failed abruptly, dominated by diagonal shear cracks; the FGC samples exhibited a mixed tension–shear mode along the weakened interface; the RC mix showed gradual lateral bulging and dispersed cracking before collapse.

**Figure 7:**
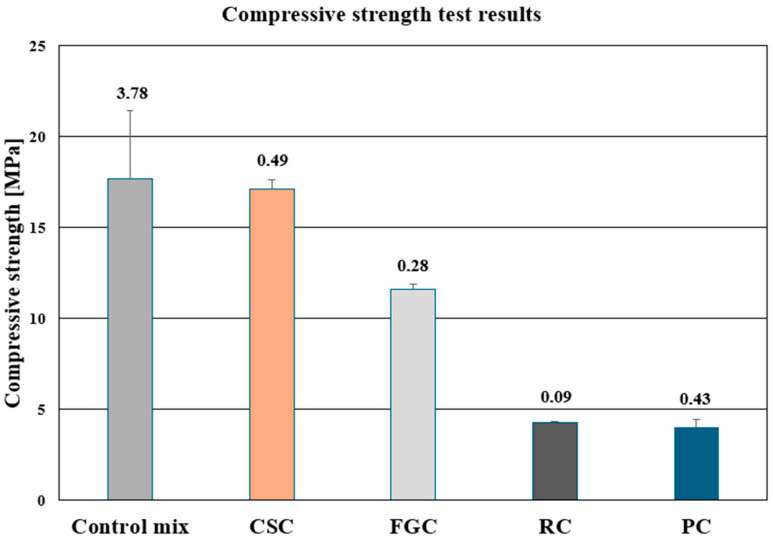
Compressive strength of the investigated mixes.

As shown in Figure 7, all mixtures display small standard deviations. The greatest variability, about 20%, appears in the RC concrete, underscoring the microstructural instability introduced by the elastic rubber aggregate. By comparison, the reference and CSC mixtures have deviations below 10%, indicating repeatable load capacity resulting from a uniform interfacial transition zone between cement paste and aggregate.

**Figure 8:**
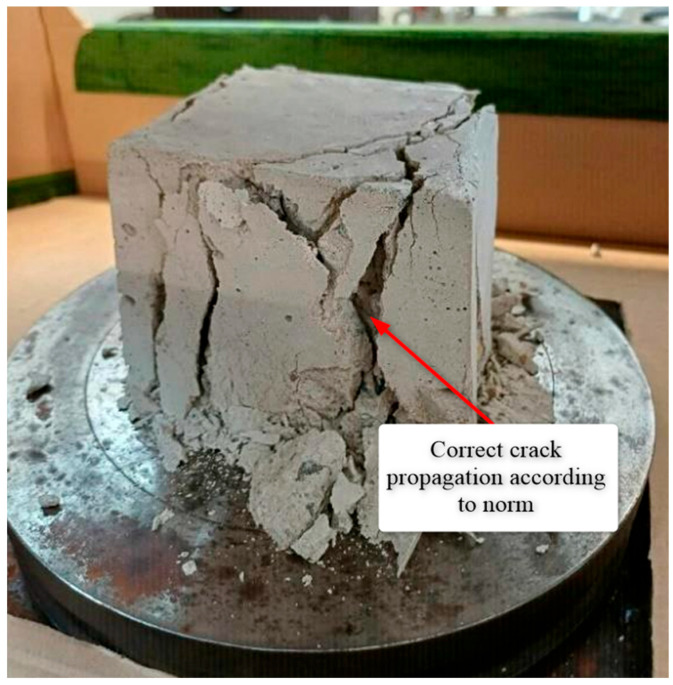
Typical failure mode of concrete specimens.

Close inspection documented in Figure 8 revealed distinctive cleavage planes that run diagonally across the entire cube cross-section in both the control and CSC specimens, a pattern consistent with brittle fracture accompanied by high energy release in the terminal failure stage. In FGC, numerous debonding planes were observed at the interface between the cement paste and the glass, confirming that the weakened transition zone governed failure rather than the strength of the binder itself. By contrast, the RC samples displayed a dispersed network of micro-cracks surrounding the rubber particles and pronounced lateral swelling before ultimate collapse, indicating that stress relaxation and energy dissipation occurred within the rubber phase.

The findings show that copper slag, owing to the hardness and angular geometry of its particles, is a fully viable substitute for sand in structural concrete, preserving-indeed in some cases enhancing-cement-matrix cohesion. By contrast, the use of glass granulate requires mix modifications, for example the addition of mineral fillers or adhesion-promoting admixtures, to compensate for the observed loss of load-bearing capacity. Incorporating both SBR rubber and polypropylene produced a pronounced drop in compressive strength, which currently limits this mix to lightly loaded elements; nevertheless, the lower density and higher deformability may prove advantageous in lightweight or energy-absorbing applications, provided that further improvements in particle adhesion, such as surface treatments or hybrid fiber reinforcement, can offset the strength reduction.

### 3.4. High Impact Test Results

The reference specimens struck by 9 × 19 mm FMJ Parabellum kinetic impactors were not perforated. Only local deformation of the impact face was observed, indicating that the impactor’s kinetic energy was insufficient to breach the material. Because full penetration did not occur, the 9 × 19 mm impactor type was excluded from subsequent testing.

When 7.62 × 54R mm impactor was used, the specimens suffered complete destruction of both the front and rear faces. The catastrophic failure prevented accurate determination of the penetration path or the impactor’s exit location; this type was therefore also removed from further analysis due to its excessive destructiveness.

**Figure 9:**
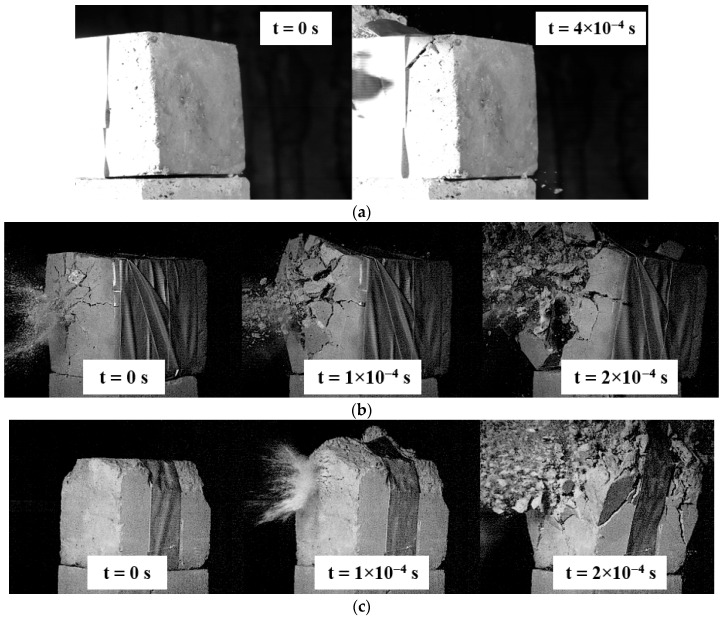
Overview of high impact tests: (**a**) impact of a 9 × 19 mm FMJ Parabellum impactor, no material penetration observed; (**b**) impact of a 7.62 × 39 mm FMJ wz. 43 PS impactor, material system damaged; (**c**) impact of a 7.62 × 54R mm FMJ soft lead-core impactor, severe specimen damage preventing trajectory observation.

The most representative and diagnostically useful results were obtained with 7.62 × 39 mm FMJ PS impactor. All specimens were cleanly perforated, displaying a clearly defined exit channel with an uninterrupted impactor trajectory and no significant loss of kinetic energy. The 7.62 × 39 mm FMJ PS impactor was therefore selected for the detailed dynamic investigations. A summary of the high impact performance for the three impactor types is presented in Figure 9.

**Figure 10:**
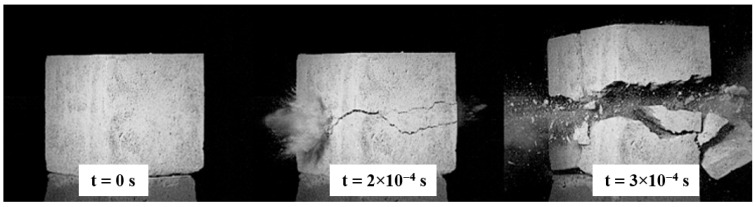
Penetration process in the specimen containing copper slag.

After the optimal type had been established, additional tests were carried out on specimens in which sand was replaced with copper slag or glass granulate. The copper-slag specimen, when struck by a 7.62 × 39 mm FMJ PS impactor, was fully perforated and then fractured into four main pieces, producing a characteristic cross-shaped crack pattern (Figure 10).

**Figure 11:**
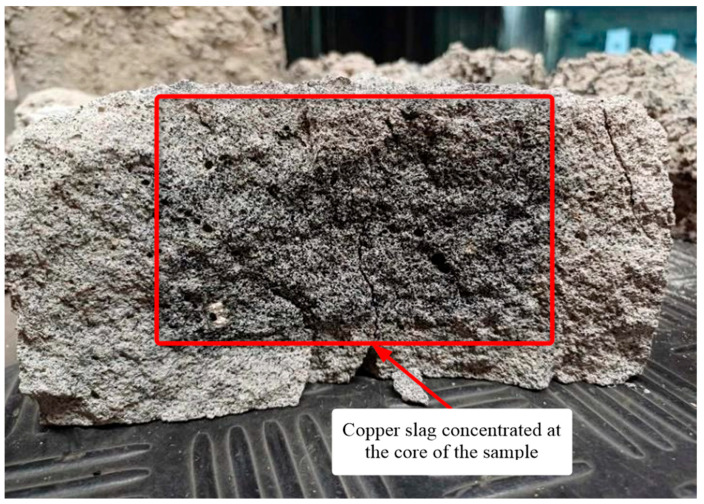
Specimen structure showing the concentration of copper slag particles.

Energy dissipation analysis was not feasible because the recording speed, 9000 frames per second, was insufficient relative to the impactor velocity. It was therefore assumed that the velocity remained nearly unchanged and that the energy loss was negligible, which in turn indicates poor dynamic performance of the copper slag specimens. In all three samples the slag formed a core concentrated in the interior of the material. This behavior may result from processing deficiencies, such as inadequate vibration or an improperly selected mix design (Figure 11).

**Figure 12:**
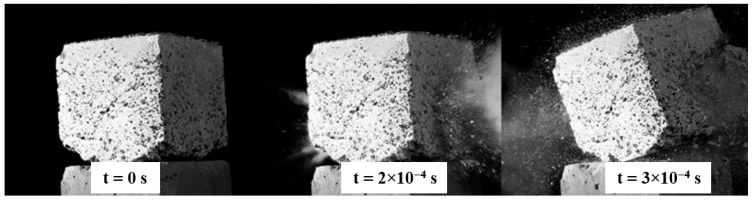
Penetration process in specimens containing SBR rubber.

The next series of specimens subjected to impact loading comprised samples containing SBR rubber, shown in Figure 12. In contrast to the previous specimen, failure did not involve global separation; instead, material was expelled from the rear face as a permanent penetration channel, that is, a Hertzian cone, formed. Owing to extensive local damage, the impactor’s trajectory could not be tracked after perforation, so the energy dissipated within the specimen could not be quantified.

**Figure 13:**
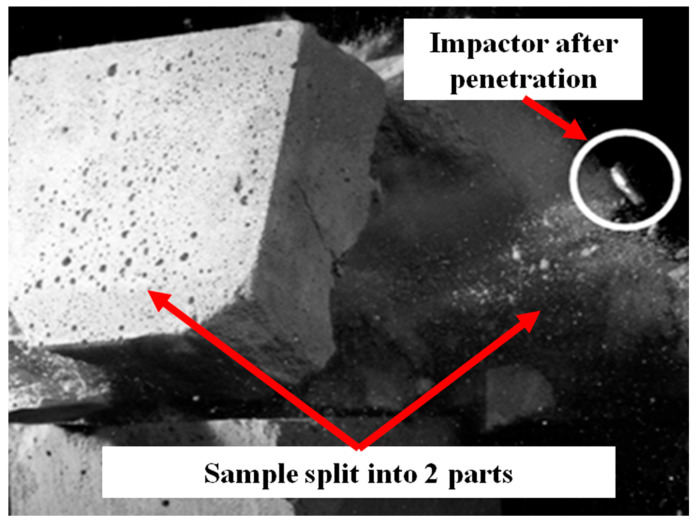
Exit hole observed in the specimen with glass granulate, indicating complete penetration.

The final specimens tested were those incorporating glass granulate, illustrated in Figure 13. Their characteristic failure mechanism was a mid-section crack that split each cube into two large pieces at the moment of impact.

Based on footage of the event, the impactor’s residual velocity was calculated, allowing the energy dissipated by the glass-granulate specimen to be determined. The sample absorbed approximately 99% of the impactor’s kinetic energy. The study therefore identified the principal failure mechanisms and the corresponding energy dissipation for each material; these results are summarized in Table 4.

Dynamic testing allowed detailed observation of the failure processes in each specimen and quantification of the impactor’s kinetic energy dissipation. The analysis showed that the failure mode varied with the type of additive incorporated into the material. These findings provide a foundation for further evaluation of the investigated materials as potential components of protective systems. 1 × 10^−4^.

### 3.5. Life Cycle Assessment

**Figure 14:**
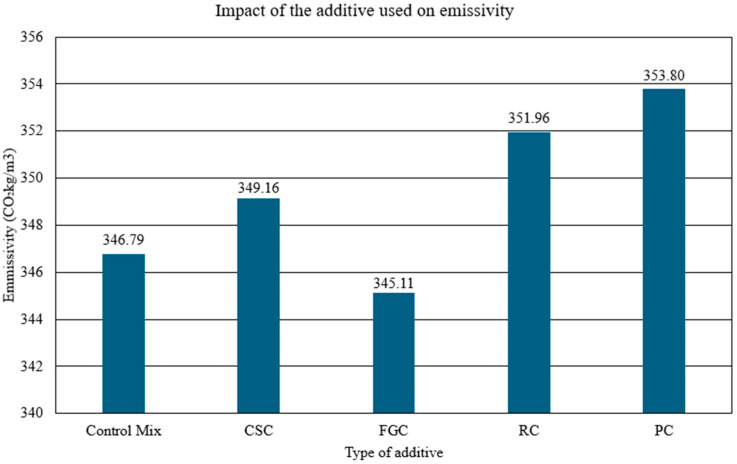
CO_2_ emissions per cubic metre of concrete for the investigated mixes.

A preliminary comparative assessment was carried out to quantify the CO_2_ emissions associated with producing three modified concretes—containing copper slag (CSC), glass granulate (FGC) and SBR rubber granulate (RC)—and to benchmark these mixes against a reference concrete prepared exclusively with natural aggregate. The evaluation followed a simplified life cycle assessment (LCA) procedure in accordance with the guidelines in refs. [40,41]. For each mix, the total CO_2_ emission per cubic metre (kg m^−3^) was estimated using specific emission factors for the principal constituents: cement, 900 kg CO_2_ t^−1^; natural aggregate, 15 kg CO_2_ t^−1^; mixing water, 0.2 kg CO_2_ t^−1^; copper slag, 50 kg CO_2_ t^−1^; glass granulate, 30 kg CO_2_ t^−1^; recycled rubber, 60 kg CO_2_ t^−1^; polypropylene, 100 kg CO_2_ t^−1^. A constant cement content of 350 kg m^−3^ was assumed, and 10% of the natural aggregate volume was replaced by the respective waste material. All calculations followed the mass-balance equation that expresses CO_2_ emission per cubic metre of concrete (1) in refs. [42,43,44,45,46]. Figure 14 compares the estimated emission values for the four mixtures.

(1) $${CO}_{2}^{total}=\sum_{i=1}^{n}\left(m_{i}\cdot EF_{i}\right)$$

where *m_i_*—mass of component *i* per 1 m^3^ of concrete (in metric tonnes); *EF_i_*—specific CO_2_ emission factor for component *i* (kg CO_2_/t).

The cost estimate for each mixture was prepared based on the mass content of individual mixture components listed in Table 2 and on the market prices of these materials.

At the specified dosage of the cement, the cost amounted to PLN 87.5 per ton in every mix. Natural aggregate, adopted as the default aggregate, was taken at PLN 55 t^−1^.

In the modified mixes, 10% of the natural aggregate was replaced by copper slag, glass granulate or SBR rubber granulate, maintaining a constant mass share of 0.1 t. Unit prices were assumed as follows: copper slag, PLN 20 t^−1^; glass granulate, PLN 220 t^−1^; recycled rubber granulate, PLN 150 t^−1^ and polypropylene, PLN 170 t^−1^. Mixing water, costed at PLN 10 t^−1^, contributed to about 5.25 PLN for each mix (Table 5).

From a sustainability standpoint, copper slag represents the most favorable compromise among emission intensity, cost and in-service performance. Glass granulate may be advantageous in applications where low self-weight and impact resistance are critical. Although SBR rubber is more expensive and exhibits lower mechanical strength, it can be beneficial in protective and damping components that demand high deformability and energy absorption.

## 4. Discussion

Replacing natural aggregate with waste-derived materials markedly influenced the behavior of concrete composites at both the micro- and macroscale. Each additive investigated—copper slag, glass granulate, and SBR rubber granulate—modified the cement–aggregate interfacial transition zone in a distinct manner, thereby governing crack-propagation patterns and the mode of mechanical energy storage within the material.

Incorporating copper slag (CSC) preserved a compressive strength comparable with that of the reference mix; this effect is attributable to the angular morphology of the particles and their favorable mechanical interlocking with the matrix. Particle sedimentation observed in specimens, accompanied by abrupt failure, indicates the need to refine compaction and homogenization procedures. Although the material achieved mechanical efficiency, it did so at the expense of reduced resistance to dynamic penetration. Similar outcomes, including diminished concrete strength when this additive is employed, have been reported elsewhere [47,48,49].

The addition of glass granulate (FGC) led to a discernible reduction in compressive strength, a result linked to the smooth and weakly adhesive glass surface. Nevertheless, the material exhibited highly favorable kinetic energy dissipation; effective impulse attenuation was recorded, rendering this variant attractive for lightweight impact-shield applications. Pronounced debonding zones suggest that enhanced grain adhesion could improve mechanical properties without compromising protective performance. Previous studies show that glass granulate additions can benefit the mechanical behavior of concrete when an appropriate mix design and a content of about 15% are employed [50,51,52,53].

Mixes containing both SBR rubber (RC) and polypropylene (PC) displayed the lowest mechanical strength in the series, owing to the particles’ low stiffness and limited adhesion to the cementitious matrix. Comparable reductions in compressive strength have been documented by other authors, who emphasize the decisive role of particle shape and size [54,55,56,57]. Despite this limitation, RC specimens responded well to impact loading: the material absorbed energy effectively (100% of projectile’s energy), and its failure mechanism indicates an ability to dissipate stresses in a manner beneficial to structural protection. The applicability of rubberized concrete should be considered primarily in protective or auxiliary elements where energy absorption and stress dissipation are prioritized over compressive capacity. Further research is required to enable its use in load-bearing structures, including the development of improved mixes with higher strength through matrix densification, fiber reinforcement, and surface modification of the SBR–cement interface, followed by verification at the structural element scale. Beyond conventional construction purposes, such concrete may also serve in strictly protective components, such as fences or energy-absorbing barriers, where reduced strength does not limit functionality. Further structural modifications—for example, surface treatment of the rubber or the inclusion of reinforcing fibers—are required to enhance mechanical resistance and material stability. As such, the polypropylene could also be treated to improve stability of the concrete. The direction of future works towards the development of numerical methods related to brittle materials will also be important, as is the case in many works [58,59].

Life cycle assessment revealed only a minor influence of the additives on overall emissions; however, employing recycled constituents positively affects the environmental balance by reducing emissions associated with other recycling processes.

## 5. Conclusions

Research confirmed that the type of aggregate is a critical determinant of the mechanical and dynamic performance of modified concretes. Depending on design objectives, industrial waste substitutes for natural aggregate can be selectively employed to enhance targeted material parameters.

The CSC mix offered the most balanced solution in terms of static load-bearing capacity, although its penetration resistance requires technological improvement. FGC concrete with glass granulate exhibited limited load capacity but a very high ability to dissipate impact energy. The RC mix, despite the lowest compressive strength, demonstrated a favorable stress dispersion mechanism and may therefore be used in protective structures where self-weight and energy absorption are paramount.

In light of these findings, further research should address

- Surface modification of mineral and polymer additives to improve adhesion to the cement matrix;
- Optimization of mixing and compaction to achieve a more homogeneous structure (particularly for CSC);
- Incorporation of dispersed reinforcement to enhance ductility and resistance to crack propagation;
- Tests on specimens conditioned in climate chambers, as well as on samples subjected to long-term storage under atmospheric conditions;
- Extension of dynamic tests to include residual velocity measurements and numerical modelling for a broader analysis of high-speed phenomena.

The results demonstrate that industrial waste can serve not only as a supplementary component of concrete mixes but also as an active constituent that tailors the protective properties of modern cement-based composites.

## Figures and Tables

**Table 1:** Physical properties of the aggregates used.

Aggregate	Specific Density (g/cm^3^)	Bulk Density (g/cm^3^)	Grain Diameter (mm)
Sand	2.62	1.70	0.05–2.00
Copper slag	3.40	1.70	0.40–1.40
Fine glass	2.50	1.40	0.90–1.50
SBR rubber	0.88	1.50	2.00–3.00
Polypropylene	0.92	1.27	1.00–1.40

**Table 2:** Constituent contents of the concrete mixes investigated.

Series	Cement [kg]	Fine Aggregate [kg]	Water [kg]	SP [%]	Fine Glass [kg]	Copper Slag [kg]	SBR Rubber [kg]	Polypropylene [kg]
Control mix	0.96	5.415	0.525	8	-	-	-	-
FGC	0.96	3.791	0.525	8	1.338	-	-	-
CSC	0.96	3.791	0.525	8	-	1.625	-	-
RC	0.96	3.791	0.525	8	-	-	1.433	-
PC	0.96	3.791	0.525	8	-	-	-	1.214

**Table 3:** Parameters of the impactors used in the tests.

Impactor Type	Mass [g]	Impactor Initial Velocity [m/s]	Kinetic Energy [J]
9 × 19 mm FMJ Parabellum	~8.0	~350	~500
7.62 × 39 mm FMJ wz. 43 PS	~7.9	~715	~2000
7.62 × 54R mm FMJ with soft lead core	~9.6	~865	~3600

**Table 4:** Summary and comparison of the firing process for the selected specimen types.

Specimen Type	Failure Mode	Energy Dissipation [%]
Copper slag	Cross-shaped fracture; specimen divided into four parts	Inconclusive
SBR rubber	Material ejected in a Hertzian-cone spall pattern	100%
Glass granulate	Specimen split into two halves (bisection)	99%

**Table 5:** Cost of mixes per ton for each material according to market prices.

Concrete Mix	Cement (PLN/t)	Natural Aggregate (PLN/t)	Substitute (PLN/t)	Water (PLN/t)	Total (PLN/t)
Ref	84	297.8	-	5. 25	387.1
CSC	84	208.5	32.5	5. 25	330.3
FGC	84	208.5	294.4	5. 25	592.1
RC	84	208.5	214.9	5. 25	512.7
PC	84	208.5	206.4	5. 25	504.1

## Data Availability

The raw data supporting the conclusions of this article will be made available by the authors on request.

## References

[1] Hamad M.A., Nasr M., Shubbar A., Al-Khafaji Z., Al Masoodi Z., Al-Hashimi O., Kot P., Alkhaddar R., Hashim K. (2021). Production of Ultra-High-Performance Concrete with Low Energy Consumption and Carbon Footprint Using Supplementary Cementitious Materials Instead of Silica Fume: A Review. Energies.

[2] Zhang H., Zhang B., Tang L., Zeng W. (2023). Analysis of Two Processing Techniques Applied on Powders from Recycling of Clay Bricks and Concrete, in Terms of Efficiency, Energy Consumption, and Cost. Constr. Build. Mater..

[3] Busch P., Kendall A., Murphy C.W., Miller S.A. (2022). Literature Review on Policies to Mitigate GHG Emissions for Cement and Concrete. Resour. Conserv. Recycl..

[4] Das S., Saha P., Prajna Jena S., Panda P. (2022). Geopolymer Concrete: Sustainable Green Concrete for Reduced Greenhouse Gas Emission—A Review. Mater. Today Proc..

[5] Sabău M., Bompa D.V., Silva L.F.O. (2021). Comparative Carbon Emission Assessments of Recycled and Natural Aggregate Concrete: Environmental Influence of Cement Content. Geosci. Front..

[6] Meng Y., Zhang C., Liu Z., Ling L., Lei J., Fang G., Luo X. (2023). Recycling of Waste Printed Circuit Boards: Effect of PCB on Aging Resistance Property of SBR Modified Asphalt. J. Build. Eng..

[7] Hiranobe C.T., Tolosa G.R., de Almeida Santos G.T., de Oliveira J.P.J., Budemberg E.R., da Silva M.J., Cabrera F.C., Job A.E., Paim L.L., Torres G.B. (2023). Recycling Waste Polyurethane from the Refrigeration Industry as Filler in SBR/NR Composites for Industrial Applications. J. Appl. Polym. Sci..

[8] Tian H., Guo Z., Pan J., Zhu D., Yang C., Xue Y., Li S., Wang D. (2021). Comprehensive Review on Metallurgical Recycling and Cleaning of Copper Slag. Resour. Conserv. Recycl..

[9] Huang L., Krigsvoll G., Johansen F., Liu Y., Zhang X. (2018). Carbon Emission of Global Construction Sector. Renew. Sustain. Energy Rev..

[10] Chen J., Wang Y., Shi Q., Peng X., Zheng J. (2021). An International Comparison Analysis of CO_2_ Emissions in the Construction Industry. Sustain. Dev..

[11] Barbhuiya S., Das B.B., Adak D. (2024). A Comprehensive Review on Integrating Sustainable Practices and Circular Economy Principles in Concrete Industry. J. Environ. Manag..

[12] Oyejobi D.O., Firoozi A.A., Fernández D.B., Avudaiappan S. (2024). Integrating Circular Economy Principles into Concrete Technology: Enhancing Sustainability through Industrial Waste Utilization. Results Eng..

[13] Shao Z., Sakai Y. (2025). Recycling Recycled Concrete Powder into Low-Carbon Construction Material through Compaction and Carbonation. Resour. Conserv. Recycl..

[14] Rosik-Dulewska C. (2015). Podstawy Gospodarki Odpadami.

[15] Xu K., Huang W., Zhang L., Fu S., Chen M., Ding S., Han B. (2021). Mechanical Properties of Low-Carbon Ultrahigh-Performance Concrete with Ceramic Tile Waste Powder. Constr. Build. Mater..

[16] Zhang L., Shen H., Xu K., Huang W., Wang Y., Chen M., Han B. (2023). Effect of Ceramic Waste Tile as a Fine Aggregate on the Mechanical Properties of Low-Carbon Ultrahigh Performance Concrete. Constr. Build. Mater..

[17] Xiao J., Cheng Z., Zhou Z., Wang C. (2022). Structural Engineering Applications of Recycled Aggregate Concrete: Seismic Performance, Guidelines, Projects and Demonstrations. Case Stud. Constr. Mater..

[18] Monteiro P.J.M., Miller S.A., Horvath A. (2017). Towards Sustainable Concrete. Nat. Mater..

[19] Al-Tarbi S.M., Baghabra Al-Amoudi O.S., Al-Osta M.A., Al-Awsh W.A., Ali M.R., Maslehuddin M. (2022). Development of Eco-Friendly Hollow Concrete Blocks in the Field Using Wasted High-Density Polyethylene, Low-Density Polyethylene, and Crumb Tire Rubber. J. Mater. Res. Technol..

[20] Sangeetha P., Annamalai V.E., Kaythry P. (2023). Durability Studies on Concrete with Partial Replacement of Cement and Coarse Aggregate by Seashell Waste. Mater. Today Proc..

[21] Thorne J., Bompa D.V., Funari M.F., Garcia-Troncoso N. (2024). Environmental Impact Evaluation of Low-Carbon Concrete Incorporating Fly Ash and Limestone. Clean. Mater..

[22] Cui K., Liang K., Jiang T., Zhang J., Lau D., Chang J. (2023). Understanding the Role of Carbon Nanotubes in Low-Carbon Concrete: From Experiment to Molecular Dynamics. Cem. Concr. Compos..

[23] Althoey F., Ansari W.S., Sufian M., Deifalla A.F. (2023). Advancements in Low-Carbon Concrete as a Construction Material for the Sustainable Built Environment. Dev. Built Environ..

[24] Das N., Nanthagopalan P. (2022). State-of-the-Art Review on Ultra High Performance Concrete—Ballistic and Blast Perspective. Cem. Concr. Compos..

[25] Rajput A., Iqbal M.A., Gupta N.K. (2018). Ballistic Performances of Concrete Targets Subjected to Long Projectile Impact. Thin-Walled Struct..

[26] Remennikov A., Gan E.C.J., Ngo T., Netherton M.D. (2019). The Development and Ballistic Performance of Protective Steel-Concrete Composite Barriers against Hypervelocity Impacts by Explosively Formed Projectiles. Compos. Struct..

[27] Das N., Nanthagopalan P. (2024). Ballistic Impact Resistance of Ultra-High-Performance Concrete (UHPC): A Comprehensive Experimental Investigation and Parametric Analysis. Int. J. Impact Eng..

[28] Wang C., Guo J., Wang X., Zhang Y., Ma Z. (2025). Dynamic Mechanical Properties and Damage Constitutive Model of High-Toughness Recycled Aggregate Concrete under High Strain Rate Impact Loads. J. Build. Eng..

[29] Afshani Z., Rofooei F.R. (2025). Experimental Study of Lightweight and Normal-Weight Concrete Slabs under Impact Loads: The Influence of Prestressing and Fiber Reinforcement. Constr. Build. Mater..

[30] Smaoui H., Trabelsi A., Kammoun Z., Aouicha B. (2023). Mechanical, Physical, Blast Waves and Ballistic Impact Resistance Properties of a Concrete Incorporating Thermally Treated PET Inclusions. Constr. Build. Mater..

[31] (2016). Test Procedures and Classification of the Effects of Weapons on Structures.

[32] Korentz J., Szmatuła F. (2020). Wpływ Dodatku Miału Gumowego SBR Na Właściwości Zapraw Cementowych. Mater. Bud..

[33] Tang B., Wu H., Wu Y.F. (2024). Evaluation of Carbon Footprint of Compression Cast Waste Rubber Concrete Based on LCA Approach. J. Build. Eng..

[34] Miah M.J., Huaping R., Paul S.C., Babafemi A.J., Sharma R., Jang J.G. (2023). Performance of Eco-Friendly Concrete Made from Recycled Waste Tire Fine Aggregate as a Replacement for River Sand. Structures.

[35] Subhani M., Ali S., Allan R., Grace A., Rahman M. (2024). Physical and Mechanical Properties of Self-Compacting Geopolymer Concrete with Waste Glass as Partial Replacement of Fine Aggregate. Constr. Build. Mater..

[36] Dynan D., Shaikh F., Derry S., Biswas W.K. (2023). Eco-Efficiency Assessment Utilizing Recycled Glass Aggregate in Concrete. Buildings.

[37] Kurniati E.O., Pederson F., Kim H.J. (2023). Application of Steel Slags, Ferronickel Slags, and Copper Mining Waste as Construction Materials: A Review. Resour. Conserv. Recycl..

[38] Al-Jabri K.S., Hisada M., Al-Saidy A.H., Al-Oraimi S.K. (2009). Performance of High Strength Concrete Made with Copper Slag as a Fine Aggregate. Constr. Build. Mater..

[39] (2013). Concrete Testing—Part 3: Compressive Strength of Test Specimens.

[40] (2006). Environmental Management—Life Cycle Assessment—Principles and Framework.

[41] (2006). Environmental Management—Life Cycle Assessment—Requirements and Guidelines.

[42] Vieira D.R., Calmon J.L., Coelho F.Z. (2016). Life Cycle Assessment (LCA) Applied to the Manufacturing of Common and Ecological Concrete: A Review. Constr. Build. Mater..

[43] Zhang Y., Luo W., Wang J., Wang Y., Xu Y., Xiao J. (2019). A Review of Life Cycle Assessment of Recycled Aggregate Concrete. Constr. Build. Mater..

[44] Andersson R., Fridh K., Stripple H., Häglund M. (2013). Calculating CO_2_ Uptake for Existing Concrete Structures during and after Service Life. Environ. Sci. Technol..

[45] Hottle T., Hawkins T.R., Chiquelin C., Lange B., Young B., Sun P., Elgowainy A., Wang M. (2022). Environmental Life-Cycle Assessment of Concrete Produced in the United States. J. Clean. Prod..

[46] Asadollahfardi G., Katebi A., Taherian P., Panahandeh A. (2021). Environmental Life Cycle Assessment of Concrete with Different Mixed Designs. Int. J. Constr. Manag..

[47] Wu W., Zhang W., Ma G. (2010). Mechanical Properties of Copper Slag Reinforced Concrete under Dynamic Compression. Constr. Build. Mater..

[48] Gupta N., Siddique R. (2020). Durability Characteristics of Self-Compacting Concrete Made with Copper Slag. Constr. Build. Mater..

[49] Wang R., Shi Q., Li Y., Cao Z., Si Z. (2021). A Critical Review on the Use of Copper Slag (CS) as a Substitute Constituent in Concrete. Constr. Build. Mater..

[50] Du H., Hwee Tan K. (2014). Waste Glass Powder as Cement Replacement in Concrete. J. Adv. Concr. Technol..

[51] Ramakrishnan K., Pugazhmani G., Sripragadeesh R., Muthu D., Venkatasubramanian C. (2017). Experimental Study on the Mechanical and Durability Properties of Concrete with Waste Glass Powder and Ground Granulated Blast Furnace Slag as Supplementary Cementitious Materials. Constr. Build. Mater..

[52] Heriyanto, Pahlevani F., Sahajwalla V. (2019). Effect of Glass Aggregates and Coupling Agent on the Mechanical Behaviour of Polymeric Glass Composite. J. Clean. Prod..

[53] Małek M., Łasica W., Kadela M., Kluczyński J., Dudek D. (2021). Physical and Mechanical Properties of Polypropylene Fibre-Reinforced Cement–Glass Composite. Materials.

[54] Grinys A., Augonis A., Daukšys M., Pupeikis D. (2020). Mechanical Properties and Durability of Rubberized and SBR Latex Modified Rubberized Concrete. Constr. Build. Mater..

[55] Prakash P.C., Gurumoorthi G., Navaneethakrishnan V., Vishvanathperumal S. (2023). Effect of Nanographene Oxide on the Mechanical Properties of EPDM/SBR Nano-Composites. Polym. Soc. Korea.

[56] Effect of SBR on Physical and Mechanical Properties of Concrete. https://www.researchgate.net/publication/334442892_Effect_of_SBR_on_Physical_and_Mechanical_Properties_of_Concrete.

[57] Qasim O.A. (2018). Experimental Investigation on Effect of SBR and Steel Fiber on Properties of Different Concrete Types. Int. J. Civ. Eng. Technol..

[58] Baranowski P., Mazurkiewicz Ł., Małachowski J., Pytlik M. (2020). Experimental Testing and Numerical Simulations of Blast-Induced Fracture of Dolomite Rock. Meccanica.

[59] Baranowski P., Kucewicz M., Gieleta R., Stankiewicz M., Konarzewski M., Bogusz P., Pytlik M., Małachowski J. (2020). Fracture and Fragmentation of Dolomite Rock Using the JH-2 Constitutive Model: Parameter Determination, Experiments and Simulations. Int. J. Impact Eng..

